# Oncology pharmacy practice in the United States: Results of a comprehensive, nationwide survey

**DOI:** 10.1177/10781552231174858

**Published:** 2023-05-16

**Authors:** Shawn P Griffin, Jessie R Signorelli, Aubrey Lasko, Benjamin J Andrick, David Doan, Shannon Hough, Grazyna Riebandt, Stephen Harnicar

**Affiliations:** 1 Department of Clinical Pharmacy Practice, School of Pharmacy & Pharmaceutical Sciences, 8788 University of California, Irvine, CA, USA; 2 Department of Pharmacy, 2348 Massachusetts General Hospital, Boston, MA, USA; 3 Enterprise Pharmacy, Center for Pharmacy Innovations & Outcomes, Danville, PA, USA; 4 Department of Pharmacy, MD Anderson Cancer Center, Houston, TX, USA; 5 10710 McKesson, Ann Arbor, MI, USA; 6 Department of Pharmacy, Clinical Pharmacy Services, 2074 Roswell Park Comprehensive Cancer Center, Buffalo, NY, USA; 7 Department of Pharmacy, 5803 Memorial Sloan Kettering Cancer Center, New York, NY, USA

**Keywords:** Oncology pharmacists, workforce, ASHP practice advancement initiative, clinical pharmacist, metrics, outcomes

## Abstract

**Introduction:** This study was designed to describe the landscape of oncology pharmacy practice at patient facing institutional healthcare organizations throughout the United States. **Methods:** The Hematology/Oncology Pharmacy Association (HOPA) Practice Outcomes and Professional Benchmarking Committee conducted a multi-organization, voluntary survey of HOPA members between March 2021 and January 2022. Four overarching domains were targeted: institutional description, job function, staffing, and training/certification. Data were evaluated using descriptive statistics. **Results:** A total of 68 responses were analyzed including 59% and 41% who self-identified their organization as academic and community centers, respectively. The median number of infusion chairs and annual infusion visits were 49 (interquartile range (IQR): 32–92) and 23,500 (IQR: 8300–300,000), respectively. Pharmacy departments reported to a business leader, physician leader, and nursing leader 57%, 24%, and 10% of the time, respectively. The median oncology pharmacy full-time equivalents was 16 (IQR: 5–60). At academic centers, 50% (IQR: 26–60) of inpatient and 30% (IQR: 21–38) of ambulatory pharmacist FTEs were dedicated to clinical activities. At community centers, 45% (IQR: 26–65) of inpatient and 50% (IQR: 42–58) of ambulatory pharmacist FTEs were dedicated to clinical activities. As many as 18% and 65% of organizations required or encouraged certification for oncology pharmacists, respectively. The median number of Board-Certified Oncology Pharmacists was 4 (IQR: 2–15). **Conclusion:** As the number of patients with cancer rises, the oncology workforce must grow to support this expanding population. These results describe the practice landscape of oncology pharmacy at US healthcare institutions to serve as a foundation for future research evaluating metrics and benchmarks.

## Introduction

Recent advances in anticancer therapeutics have changed the treatment landscape for patients. Yet, as the US population ages, the number of patients with cancer is expected to rise. Recently, the Centers for Disease Control and Prevention predicted the total number of incident cancer cases to increase by almost 50% between 2015 and 2050.^1^ In response, the oncology workforce must grow to support the expanding patient population. A 2014 investigation projected there would be a shortage of nearly 2400 oncologists by 2025.^2^ Therefore, the need to expand the nonphysician oncology workforce in this potential care vacuum is paramount.

Pharmacists are uniquely positioned to meet patient care needs of the growing population of people living with cancer. In a position paper, the Hematology/Oncology Pharmacy Association (HOPA) highlighted the importance of pharmacists in the delivery of care for individuals living with cancer.^3^ This was rooted in historical evidence that oncology pharmacists have positively impacted patient care over the previous decades.^4,5^ The HOPA position paper envisions the role of oncology pharmacists further expanding to include practices across a variety of settings, expansion of medication therapy management programs, development of independent prescribing protocols, and more, to address the evolving landscape of cancer care and increased need for oncology workforce.

Subsequently, the role and value of the oncology pharmacists has accelerated both nationally and internationally.^6,7^ Oncology pharmacists continue to demonstrate leadership in developing clinical and operational guidelines across a variety of pharmacy practice settings and subspecialties.^8–12^ Practice sites for oncology pharmacists vary including inpatient units, ambulatory clinics, infusion centers, specialty pharmacies, investigational drug services (IDS), managed care institutions, as well as practice management and leadership. Additionally, institutional practice may vary from large academic medical centers to local community cancer practices in rural or urban communities. The recent expansion of telehealth pharmacy services increases the reach of pharmacists to provide care as well as added convenience and access for patients. The diversity of pharmacy practice in oncology has grown in parallel to meet the expansion of care delivery services and settings for patients with cancer.

Oncology pharmacists receive focused and intensive training. In addition to receiving a Doctor of Pharmacy degree, individuals can undergo 2 years of additional postgraduate residency training accredited by the American Society of Health-System Pharmacists (ASHP). The postgraduate year 2 (PGY2) oncology residencies have goals and objectives developed jointly by HOPA and ASHP to create standards for extensive training and competencies for oncology pharmacists. Thus, graduates of such programs are prepared to provide effective pharmacotherapy services to patients with cancer. Finally, pharmacists practicing in hematology/oncology can attain formal board certification and nationwide recognition as Board-Certified  Oncology Pharmacist (BCOP). Ignoffo and colleagues recently concluded that oncology pharmacists are qualified and well-equipped to aid the oncology workforce in patient care.^13,14^

A recent systemic literature review of over 400 articles since 1950 found that published work on oncology pharmacy practice centered around four key areas of value: clinical care, patient education, informatics, and cost savings.^15^ However, the specific outcomes to measure each area were heterogeneous. To understand the oncology pharmacy practice landscape, Ignoffo and colleagues also recently conducted interviews with oncology pharmacy leadership at 20 intuitions across the United States.^16^ The survey evaluated criteria to forecast the workforce needs and practices. A common theme among metrics reported was difficulty in obtaining data. The study additionally identified limitations for future including, pharmacist involvement in credentialing, quality measures, and value-based reimbursement systems. Often, institutions utilize cost-avoidance studies to demonstrate the impact of pharmacist interventions on patient care. However, a recent systematic literature evaluation identified that this methodology used in pharmacy literature should be continually refined.^17^

There is no nationwide description of oncology pharmacy practice at healthcare institutions. A baseline understanding of current oncology pharmacy practice is needed, specifically the number of employees, types of practice models, and commonly utilized practice metrics. Such a foundation is necessary for discussions and research to determine meaningful metrics for inter-institutional and intra-institutional benchmarking. Therefore, we designed a nationwide survey to investigate and describe the current oncology pharmacy practice landscape.

## Methods

This study was completed via a multi-organization, voluntary survey to assess the national landscape of oncology pharmacy practice. The HOPA Practice Outcomes and Professional Benchmarking Committee (POPBC) began questionnaire development in 2019 based on committee charges to benchmark current US oncology pharmacy practice. HOPA is a nonprofit, education-based organization formed in 2004 to help oncology and hematology pharmacy practitioners and their associates provide the best possible cancer care. HOPA serves more than 3000 members in the fields of oncology pharmacy by supporting research, providing education, encouraging professional development, developing best practice standards, and advocating for health policy issues that improve patient care.

Survey questions were developed to target four overarching domains: Institutional Description, Job Function, Staffing, and Training and Certification. The survey was beta tested with volunteers from the committee for edits and length of time to completion. After initial testing, the finalized survey was composed of 33 questions, which included a variety of question/statement types: closed-ended (i.e. Yes/No), multiple-choice, open-ended, and optional statements. The entire survey is provided in the Supplemental material.

### Study design

The institution review board at the University of California, Irvine determined that this study was exempt from review and did not require informed consent. The POPBC identified potential target organizations through the HOPA membership database. This database is populated with membership data provided during the initial sign-up and annual renewal process. For the purposes of this study, an organization was defined as an entity providing healthcare in at least one of the following settings: inpatient, outpatient, infusion, or satellite facilities. Organizations in the pharmaceutical industry, pharmacy benefit managers, schools or colleges of pharmacy, health-related technology companies, and international organizations were excluded. Based on historical survey response rate data, we anticipated a successful response rate to be 20%. The questionnaire platform was administered via SurveyMonkey® (SurveyMonkey®, San Mateo, CA) by email to recipients at eligible organizations. Multiple individuals within the same organization were emailed the survey link to increase the chance it reached those who would best be able to answer the questions. To ensure data integrity, respondents were instructed to work with colleagues to complete the survey in full and only submit one response for each organization. The survey was sent in four phases to different organizations to maximize response rate. Surveys were sent between March 2021 and January 2022. Respondents were requested to complete the survey within 2 weeks.

In circumstances where a recipient responded to the survey more than once, responses from each questionnaire were compiled if there were no discrepancies between the surveys. In circumstances where multiple recipients from within the same organization responded to the survey, the questionnaire with the most complete responses was accepted. Discordant survey responses to questions from either the same individual or more than one individual from the same organization were excluded from the results. Because not all questions were answered by each participant, number of responders for each question are depicted in tables. Readers may contact the corresponding author to inquire or request access to the dataset used.

### Study objective

The primary objective was to describe the landscape of oncology pharmacy practice at healthcare organizations throughout the United States.

### Statistical analysis

The data collected were analyzed through Microsoft Excel (version 16.0.15028.20160, Microsoft Corporation, Redmond, WA). Descriptive statistics were used to evaluate data. For survey responses that the committee deemed were implausible, the implausible data point was considered missing (e.g. reporting 0 infusion chairs but 1000 infusion visits). Results were further split into academic and community centers to allow end-users to focus on data most applicable to their respective institutions. This classification was conducted using self-reported data from each respondent. For those who described themselves as a hybrid organization, POPBC membership evaluated the features of the organization and re-categorized as either academic or community. In instances where a percentage was calculated, the denominator was the total number of respondents for that survey question; blank responses were not included in these calculations. For continuous variables, answers were reported with a median, interquartile range, and range (Q0 = 0%, Q1 = 25%, Q3 = 75%, Q4 = 100%) to allow for a more complete description of data distribution.

## Results

Across the four phases of distribution, the survey was sent to 1280 individuals representing 650 distinct organizations. A total of 89 responses were received. After multiple responses from a single individual and duplicate responses from the same organizations were removed, 68 (10%) responses remained. Of these, 40 (59%) and 28 (41%) self-identified their organizations as academic and community centers, respectively.

### Organizational description

Among all centers providing a response, the median number of infusion chairs reported was 49 (Q0: 9, Q1: 32, Q3: 92, Q4: 350) and the median number of annual infusion visits were 23,500 (Q0: 200, Q1: 8300, Q3: 51,700, Q4: 300,000). For academic centers, the median number of infusion chairs reported was 78 (Q0: 20, Q1: 47, Q3: 153, Q4: 350) and the median number of annual infusion visits was 48,200 (Q0: 2000, Q1: 25,100, Q3: 64,500, Q4: 300,000). For community centers the median number of infusion chairs reported was 31 (Q0: 9, Q1: 15, Q3: 43, Q4: 140) and the median number of annual infusion visits was 12,000 (Q0: 200, Q1: 5000, Q3: 15,700, Q4: 43,000). Among academic centers, 68% reported having 50 or more dedicated inpatient oncology beds and 6% of community centers reported the same. For those centers reporting oncology bed utilization (N = 32), 83% of all centers, 96% academic centers, and 50% community centers had >75% of inpatient oncology beds filled. No academic centers had <25% of inpatient oncology beds filled while 25% of community centers reported such.

Among all organizations providing a response (N = 66), most pharmacy departments reported up through a pathway led by a business leader (57%) as opposed to a physician leader (24%) or a nursing leader (10%). No academic centers ultimately reported up to a nursing leader and 25% of community centers reported to a nursing leader. Most organizations operated exclusively under a fee-for-service model (75%). While 32% of academic centers reported participating in an alternative payment model of some type, no community centers reported similar participation. There was wide variation in payer mix with a median of 40% (Q0: 4, Q1: 30, Q3: 50, Q4: 70) being attributed to government payers among all organizations. Additional results related to organizational descriptions, including number of survey responses for each variable not reported in this section, are depicted in Tables 1 and 2.

**Table 1.** Institutional description.

Descriptor, no. (%)	All centers	Academic centers	Community centers
	N = 68	N = 40	N = 28
Provider oncology visits, total responses	38(56)	22(55)	16(57)
Visits, thousands^a^	25(2.5, 9.1, 147, 1300)	133.7(4.5, 26.5, 238, 1300)	12.8(2.5, 3.8, 19.7, 58)
Infusion visits, total responses	43(63)	26(65)	17(61)
Visits, thousands^a^	23.5(0.2, 8.3, 51.7, 300)	48.2(2, 25.1, 64.5, 300)	12(0.2, 5, 15.7, 43)
Infusion chairs, total responses	48(71)	30(75)	18(64)
Chairs^a^	49(9, 31.5, 91.8, 350)	77.5(20, 46.5, 153, 350)	31(9, 14.8, 43, 140)
Inpatient beds, total responses	48(71)	31(78)	17(61)
1–99	3(6)	1(3)	2(12)
100–249	7(15)	3(10)	4(24)
250–499	7(15)	2(6)	5(29)
500–999	18(38)	13(42)	5(29)
Over 1000	13(27)	12(39)	1(6)
Inpatient oncology beds, total responses	48(71)	31(78)	17(61)
0–49	26(54)	10(32)	16(94)
50–99	8(17)	7(23)	1(6)
100–249	12(25)	12(39)	0
250–499	0	0	0
500–999	2(4)	2(6)	0
Payment model, total responses	32(47)	19(48)	13(46)
Fee for service	24(75)	15(79)	9(69)
Fee for service and value or outcome-based	5(16)	3(16)	2(15)
Value or outcome-based	1(3)	0	1(8)
Other	2(6)	1(5)	1(8)
Participation in oncology care or alternative	6(19)	6(32)	0
Payor mix, total responses	32(47)	19(48)	13(46)
Federal: Medicare/Medicaid^a^	40(5, 30, 50, 70)	35(5, 27.5, 48, 67)	43(10, 35, 55, 70)
Federal: VA/DOD^a^	1.5(0, 0, 5, 20)	1(0, 0, 5, 20)	2(0, 0, 5, 10)
Commercial^a^	33(15, 25, 41.3, 60)	34(15, 27.5, 45, 60)	30(15, 25, 35, 60)
Commercial: Medicare/Medicaid^a^	20(0, 9.8, 30, 47)	10(0, 8.5, 21, 47)	25(0, 10, 30, 40)

^a^Results reported as median (0%, 25%, 75%, 100% quartile).

DOD: department of defense; VA: veterans affairs.

**Table 2.** Oncology practice description.

Descriptor, no. (%)	All centers	Academic centers	Community centers
	N = 68	N = 40	N = 28
Nonpharmacy oncology FTEs, total responses	32(47)	18(45)	14(50)
Physicians^a^	20(0, 5, 140, 1800)	99(0, 25, 183, 1800)	7(3, 4.3, 11.5, 250)
Advanced practice providers^a^	10(0, 4, 73, 964)	35(0, 9.5, 138, 964)	4(0, 3, 6.5, 350)
Nurses^a^	50(0, 23.8, 157, 9999)	108.5(0, 38.8, 600, 9999)	30(5, 16.3, 46.3, 2500)
Oncology practice sites, total responses	68(100)	40(100)	28(100)
Main campus outpatient clinic	62(92)	37(93)	25(89)
Offsite/satellite clinic	40(59)	28(70)	12(43)
Main campus infusion center	61(90)	39(98)	22(79)
Offsite/satellite infusion center	43(63)	30(75)	13(46)
Inpatient facility	65(96)	39(98)	26(93)
Retail pharmacy	50(74)	32(80)	18(64)
Retail specialty pharmacy	50(74)	35(88)	15(54)
Home infusion	22(32)	17(43)	5(18)
Oncology multidisciplinary team composition, total responses	35(51)	20(50)	15(54)
Medical scribes	9(26)	7(35)	2(13)
Patient navigators	30(86)	16(80)	14(93)
Case managers	24(69)	16(80)	8(53)
Medical assistants	27(77)	17(85)	10(67)
Medication access coordinators	18(51)	14(70)	4(27)
Social workers	32(91)	19(95)	13(87)
Dietitians	30(86)	18(90)	12(80)
Research study coordinators	31(89)	19(95)	12(80)
Financial advocates	26(74)	14(70)	12(80)

^a^Results reported as median (0%, 25%, 75%, and 100% quartile).

FTEs: full-time equivalents.

### Organizational staffing and job functions

The median oncology pharmacy full-time equivalents (FTEs) reported was 16 (Q0: 1, Q1: 5, Q3: 60, Q4: 570) among all centers and 59 (Q0: 1, Q1: 24, Q3: 88, Q4: 570) and 6 (Q0: 1, Q1: 4, Q3: 10, Q4: 29) for academic and community centers, respectively. For academic centers there was a median of 13 (Q0: 1, Q1: 6, Q3: 25, Q4: 340) inpatient oncology pharmacist, 23 (Q0: 1, Q1: 11, Q3: 30, Q4: 175) ambulatory oncology pharmacist, and 29 (Q0: 1, Q1: 13, Q3: 41, Q4: 220) oncology pharmacy technician FTEs. Inpatient pharmacists spent 50% (Q0: 1%, Q1: 26%, Q3: 60%, Q4: 90%) while ambulatory pharmacists spent 30% (Q0: 1%, Q1: 21%, Q3: 38%, Q4: 52%) of time on clinical activities in academic centers. For community centers there was a median of 2 (Q0: 0, Q1: 1, Q3: 3, Q4: 16) inpatient oncology pharmacist, 3 (Q0: 1, Q1: 2, Q3: 7, Q4: 13) ambulatory oncology pharmacist, and 4.5 (Q0: 1, Q1: 3, Q3: 6, Q4: 16) pharmacy technician FTEs. In these community centers, inpatient oncology pharmacists spent a median of 45% (Q0: 0%, Q1: 26%, Q3: 65%, Q4: 100%) and ambulatory oncology pharmacist spent a median of 50% (Q0: 20%, Q1: 42%, Q3: 58%, Q4: 100%) of their time engaged in clinical activities. Academic centers reported employing a median of 99 (Q0: 0, Q1: 25, Q3: 183, Q4: 1800) and 108.5 (Q0: 0, Q1: 39, Q3: 600, Q4: 9999) oncology-specific physician and nurse FTEs, respectively. Community centers employed a median of 7 (Q0: 3, Q1: 4, Q3: 12, Q4: 250) and 30 (Q0: 5, Q1: 16, Q3: 46, Q4: 2500) physician and nurse FTEs, respectively. Additional results, including number of survey responses for each variable, are depicted in Tables 3 and 4.

**Table 3.** Pharmacy staffing and job function.

Descriptor, no. (%)	All centers	Academic centers	Community centers
	N = 68	N = 40	N = 28
Oncology pharmacy FTEs, total responses	32(47)	18(45)	14(50)
Total FTEs^a^	16 (1, 5, 60, 570)	59 (1, 23.8, 88.2, 570)	6 (1, 4.3, 10.1, 29)
Inpatient pharmacist^a^	6(0, 1.8, 15.3, 340)	13(1, 6.3, 24.6, 340)	2 (0, 0.6, 3, 16)
Ambulatory pharmacist^a^	10.3(1, 3, 25, 175)	23(1, 10.5, 29.5, 175)	3 (1, 2, 6.5, 13.4)
Retail pharmacist^a^	1(0, 0, 3.8, 35)	3(0, 1, 5, 35)	0 (0, 0, 0, 7)
IDS pharmacist^a^	1(0, 0, 3.3, 19)	2(0.25, 2, 5, 19)	0 (0, 0, 0.7, 3)
Pharmacy technicians^a^	10.9(1, 3.9, 29.5, 220)	29(1, 13, 41, 220)	4.5 (1, 3, 6.1, 16)
Oncology pharmacist activities, total responses	34(50)	19(48)	15(54)
Drug prior authorizations	22(65)	15(79)	7(47)
Patient education	29(85)	18(95)	11(73)
Patient assistance to access oncology drugs	21(62)	14(74)	7(47)
Transitional care	22(65)	17(89)	5(33)
Medication reconciliation	24(71)	18(95)	6(40)
Other	5(15)	1(5)	3(20)
Oncology pharmacy technician activities, total responses	34(50)	19(48)	15(54)
Drug prior authorizations	12(35)	8(42)	4(27)
Patient assistance to access oncology drugs	21(62)	16(84)	5(33)
Transitional care	4(12)	4(21)	0
Medication reconciliation	9(26)	7(37)	2(13)

^a^Results reported as median (0, 25, 75, 100% quartile).

FTEs: full-time equivalents; IDS: investigational drug services.

**Table 4.** Division of oncology pharmacy services.

	Academic centers		Community centers	
	N = 18		N = 14	
Role	Percentage in operational role, median (0%, 25%, 75%, 100% quartile)	Percentage in clinical role, median (0%, 25%, 75%, 100% quartile)	Percentage in operational role, median (0%, 25%, 75%, 100% quartile)	Percentage in clinical role, Median (0%, 25%, 75%, 100% quartile)
Inpatient pharmacist	50(0, 26.3, 59.5, 80)	50(1, 26.25, 60, 90)	50(0, 6.3, 67.5, 75)	45(0, 26.3, 65, 100)
Ambulatory pharmacist	70(1, 52.5, 74, 80)	30(1, 21.3, 38.3, 52)	50(0, 42.5, 57.5, 80)	50(20, 42.5, 57.5, 100)
Retail/specialty pharmacist	80(0, 40, 90, 100)	20(0, 0, 20, 100)	0(0, 0, 10, 90)	0(0, 0, 5, 80)
IDS pharmacist	70(1, 50, 87.5, 100)	28.5(0, 3.3, 47.5, 60)	0(0, 0, 50, 70)	0(0, 0, 45, 85)
Pharmacy technician	94(1, 90, 100, 100)	3(0, 0, 10, 20)	95(0, 80, 100, 100)	5(0, 0, 20, 100)

IDS: investigational drug services.

### Organizational training

The median number of BCOP among all organizations was 4 (Q0: 0, Q1: 2, Q3: 15, Q4: 80) with academic centers reporting a median of 13 (Q0: 1, Q1: 6, Q3: 19, Q4: 80) and community centers reporting a median of 2 (Q0: 0, Q1: 1, Q3: 3, Q4: 18). Among all centers, 18% and 65% either required or encouraged certification for oncology pharmacists, respectively. Academic centers reported a median of 32% of state boards of pharmacy and 58% of their individual organizations having a credentialing and privileging system for oncology pharmacists. Community centers indicated that no state boards of pharmacy and 13% of individual organizations had a credentialing and privileging system for oncology pharmacists. These results are shown in Table 5. Of the 50 responses pertaining to training programs, 96% of centers reported training pharmacy students or interns. Of the academic centers providing a response (N = 32), 88% trained PGY1 residents, 94% PGY2 residents, and 19% pharmacy fellows. Of the community centers (N = 18), 61% trained PGY1 residents, 28% trained PGY2 residents, and none trained pharmacy fellows. There was a median of 2 (Q0: 0, Q1: 1, Q3: 3, Q4: 6) and 0 (Q0: 0, Q1: 0, Q3: 1, Q4: 7) oncology PGY2 pharmacy residents at academic and community centers, respectively. Related results, including number of survey responses for each variable, are shown in Tables 5 to 7. Of all respondents, 48%, 62%, and 60% reported that pharmacists provided training for advanced practice providers, nurses, and fellow physicians, respectively. The full results are shown in Table 8.

**Table 5.** Oncology pharmacist credentialing and training.

Descriptor, no. (%)	All centers	Academic centers	Community centers
	N = 68	N = 40	N = 28
Oncology pharmacist advanced training, total responses	33(49)	19(48)	14(50)
BCOP certification^a^	4(0, 2, 15, 80)	13(1, 6, 19, 80)	2(0, 1, 2.8, 18)
Other BCPS certification^a^	1(0, 0, 5, 30)	5(0, 1, 6.5, 30)	1(0, 0, 1, 5)
PGY1 residency training^a^	5(0, 2, 17, 120)	15(0, 6.5, 23.5, 120)	1.5(0, 0.25, 2.8, 13)
PGY2 residency training^a^	3(0, 0, 14, 110)	13(0, 4.5, 15, 110)	0(0, 0, 1, 7)
Pharmacy residents at center, total responses	50(74)	32(80)	18(64)
Number of PGY1 residents^a^	4(0, 2, 9.8, 44)	6(0, 3.8, 11.3, 44)	2.5(0,0, 3.8, 15)
Number of PGY2 residents, all specialties^a^	3(0, 0, 8, 22)	7.5(0, 2.8, 11.3, 22)	0(0, 0, 2, 4)
Number of PGY2 oncology residents^a^	1(0, 0, 2, 6)	2(0, 1, 3, 6)	0(0, 0, 0, 1)
Credentialing and privileging programs, total responses	34(50)	19(48)	15(54)
From state board	6(18)	6(32)	0
From organization	13(38)	11(58)	2(13)
Requirement for advanced pharmacy practice certification, total responses	34(50)	19(48)	15(54)
Certification required	6(18)	5(26)	1(7)
Certification encouraged	22(65)	13(68)	9(60)
Not required or encouraged	6(18)	1(5)	5(33)

^a^Results reported as median (0%, 25%, 75%, and 100% quartile).

BCOP: Board-Certified Oncology Pharmacist; BCPS: Board-Certified Pharmacotherapy Specialist; PGY1: postgraduate year 1; PGY2: postgraduate year 2.

**Table 6.** Training requirements for oncology pharmacists by center type.

	Academic centers					
Position, no. (%)	PGY1	PGY2	BCOP	Other BCPS	Other certification	None required
Inpatient pharmacist, N = 17	4(24)	3(18)	2(12)	1(6)	1(6)	12(71)
Infusion pharmacist, N = 16	3(19)	1(6)	0	0	1(6)	13(81)
Retail/specialty pharmacist, N = 16	1(6)	0	0	0	0	15(94)
IDS pharmacist, N = 16	3(19)	0	0	0	0	13(81)
Inpatient clinical pharmacist specialist, N = 17	4(24)	7(41)	5(29)	0	0	7(41)
Ambulatory clinical pharmacist specialist, N = 17	5(29)	7(41)	5(29)	0	0	6(35)
Community centers					
Inpatient pharmacist, N = 10	0	0	1(10)	0	0	9(90)
Infusion pharmacist, N = 10	0	0	1(10)	0	0	9(90)
Retail/specialty pharmacist, N = 10	0	0	1(10)	0	0	9(90)
IDS pharmacist, N = 10	0	0	1(10)	0	0	9(90)
Inpatient clinical pharmacist specialist, N = 10	1(10)	1(10)	1(10)	0	0	7(70)
Ambulatory clinical pharmacist specialist, N = 10	1(10)	1(10)	1(10)	0	0	7(70)

BCOP: Board-Certified Oncology Pharmacist; BCPS: Board-Certified Pharmacotherapy Specialist; IDS: investigational drug services; PGY1: postgraduate year 1; PGY2: postgraduate year 2.

**Table 7.** Privileges through credentialing and privileging for oncology pharmacists.

	Inpatient		Ambulatory	
Privilege, no. (%)	Academic centers	Community centers	Academic centers	Community centers
	N = 11	N = 2	N = 11	N = 2
Disease state management	5(45)	0	6(55)	0
Physician exam	1(9)	0	3(27)	0
Medication safety monitoring	4(36)	1(50)	6(55)	1(50)
Evaluate need and order vaccinations	3(27)	0	5(45)	0
Administer vaccinations	0	0	1(9)	0
Administer other medications	2(18)	0	1(9)	0
Collaborative practice agreements	6(55)	1(50)	9(82)	2(100)
Pharmacy to dose protocols	9(82)	1(50)	6(55)	2(100)
Prescriptive authority	8(73)	0	7(64)	1(50)
Dose modifications	7(64)	0	7(64)	2(100)
Ordering labs	6(55)	1(50)	6(55)	2(100)
None	0	1(50)	1(9)	0

**Table 8.** Nonpharmacy training conducted by oncology pharmacists.

Role group, no. (%)	All centers	Academic centers	Community centers
	N = 50	N = 32	N = 18
Advanced practice providers	24(48)	22(69)	2(11)
Medical residents	27(54)	24(75)	3(17)
Medical students	22(44)	21(66)	1(6)
Nursing students	11(22)	10(31)	1(11)
Nurses	31(62)	23(72)	8(44)
Oncology/hematology fellows	30(60)	29(91)	1(11)

## Discussion

This is the first comprehensive, nationwide survey that describes the practice of oncology pharmacy at healthcare institutions in the United States. The results included herein lay the foundation for future practice-related metric and benchmarking research to evaluate and advance pharmacy practices at cancer centers across the country. The purpose of the data is not to evaluate the best measure of productivity, determine the ideal services provided by oncology pharmacy departments, or compare academic and community centers. Rather, the study results should be used to inform future evaluation and discussion of oncology pharmacy practice.

A few response areas stood out as key descriptions of oncology pharmacy or as specific areas for future evaluation and growth. There was a wide range of oncology pharmacy FTEs reported by survey respondents from as few as one to as many as 570, likely attributable to the varying size of individual institutions and the services devoted specifically to oncology care. Several survey respondents, including the top of the range for number of FTEs, were large, standalone cancer centers. The highest number of oncology pharmacist FTEs was dedicated to the ambulatory infusion or clinic setting. This is consistent with current trends in oncology care as most treatments occur in the outpatient setting.^18–20^ While there was a similar division of pharmacist FTEs in the inpatient and clinic/infusion setting among academic and community institutions, there were more FTEs dedicated to oncology pharmacists in the retail/specialty and IDS settings at academic institutions. These two areas represent opportunities for growth and expansion of oncology pharmacy practice, particularly at community centers. As the rate of oral oncolytic use increases, dispensing pharmacists of these agents provide pharmacotherapy expertise for both patients and providers while also making an important impact on adherence and adverse effect management.^9,21–23^

As oncology pharmacy continues to grow in both the number of FTEs and types of services that pharmacists provide, an increasing importance is placed on pharmacist training and competence.^24^ This is particularly true as many of the areas of growth are more clinical versus operational in nature. Based on the survey results, most organizations encourage an advanced pharmacy practice certification and/or proof of competence for their oncology pharmacists. PGY2 oncology pharmacy residency completion and BCOP requirements were most common in clinical pharmacist specialist roles in the inpatient and ambulatory settings compared to general inpatient or ambulatory infusion pharmacist roles. The IDS pharmacist and ambulatory retail/specialty pharmacist roles had the fewest number of required training or certification, which is likely associated with the smaller number of residency programs and certifications in these practice areas.^25^ Although the median number of BCOP-certified pharmacists was higher at academic centers, the proportion to the total number of oncology pharmacy FTEs was similar between academic and community settings. There was a comparatively low number of other board certifications, which underscores the emphasis on oncology in these roles. In line with pharmacy specific training, many respondents indicated that pharmacists were involved in the training of nonpharmacy, multidisciplinary training. This highlights the role pharmacists, as medication experts play in the education of all oncology professionals.

While residency training and board certification indicate a certain level of specialized knowledge, credentialing and privileging systems can provide a more delineated assessment of services and activities oncology pharmacists provide. Most respondents overall indicated that their state board of pharmacy does not have a credentialing and privileging system for oncology pharmacists. The number of organizations that have an individual credentialing and privileging system in place correlated with the number of oncology pharmacy FTEs, BCOP certification, and PGY2 training.^26^ Implementing institutional credentialing and privileging through collaborative practice agreements can decrease costs, prevent chemotherapy errors, and increase staff satisfaction.^27–29^ Clinical activities, considerations, and a framework for implementing credentialing and privileging have been described elsewhere, and this continues to be an area of growth for oncology pharmacy.^26,30^

Overall, pharmacy technicians represented a large proportion of the total oncology pharmacy FTEs (median 11 (Q0: 1, Q1: 4, Q3: 30, Q4: 220)) out of a total pharmacy FTE of median 16 (Q0: 1, Q1: 5, Q3: 60, Q4: 570). However, there appeared to be a much higher proportion of total oncology FTEs devoted to pharmacy technicians at community as compared to academic institutions. Pharmacy technician FTEs were nearly completely devoted to operational activities. Given the expanding clinical role of the pharmacist, some of the reported pharmacist activities could be delegated to pharmacist technicians. A median of 71%, 65%, and 62% of centers reported pharmacists are involved in medication reconciliation, prior authorizations, and assistance with accessing oncology drugs, respectively. Pharmacy technicians were reported to be involved in 26%, 35%, and 62% of medication reconciliation, prior authorizations, and assistance with accessing oncology drug programs. Delegating roles such as medication reconciliation and prior authorizations could create more time for pharmacists to expand practice while increasing job satisfaction for other role groups looking for more direct patient care.^10^ Slightly more than half of centers reported employing medical access coordinators. Increased use of this discipline, in addition to expanding pharmacy technician roles, may also allow pharmacists to focus and prioritize other activities.

Given the design of this study, response rate, response bias, and participant misreporting were potential limitations. Our overall response rate (10%) was slightly lower than initially predicted, although it is similar to what has been described in previous pharmacy surveys.^31^ To obtain as diverse a sample as possible, all those meeting our organization criteria from the HOPA membership were distributed the survey via email. No additional communication or marketing was performed. The length and specific details required for the survey may also have contributed to the lower-than-expected response rate or high number of blank responses for certain survey questions. Much of the information would not be readily available to general practicing pharmacy members of the organization.

The geographic distribution (e.g. northeast, southwest, etc.) of the respondents was similar to the larger sample (the 650 distinct organizations sent the survey) as a whole, confirming that the results reported likely represent the entire United States. Center type (i.e. academic versus community) in the survey was self-identified by individual respondents and therefore not available for the larger sample size. However, the distribution is in line with the practice sites reported by the HOPA membership as a whole where those practicing at academic medical centers outnumber those at community hospitals by a factor of 1.8 to 1. While not intentional, there were no standalone retail pharmacies, specialty pharmacies, or home infusion pharmacies among the survey respondents.

It is possible that for certain survey answers reported may not truly represent the entire population due to a small sample size. This is especially true for those questions with the fewest number of responses or questions that were only to be answered based on affirmative responses to previous questions. To be transparent about this possibility, the number of responses has been included for each survey variable. Furthermore, given the purpose of our study—to serve as an initial landscape of oncology practice and foster future discussion and research—it is important to include all survey results even with the given limitations.

Participant misreporting is a real possibility given the complexity of information collected in this survey and the lack of a uniform standard among organizations for reporting certain metrics. To combat this, several of the survey questions utilized ranges for responses in areas that may change from time to time (e.g. number of oncology beds) or may be prone to estimation. Additionally, results reported include medians and ranges whenever possible to limit the impact of one outlier.

## Conclusion

Oncology pharmacy practice encompasses the entire continuum of care and oncology pharmacists are present in the inpatient, clinic/infusion, specialty/retail, and IDS areas. The results of this study describe the practice landscape of oncology pharmacy in these settings in the United States and serve as a foundation for future research evaluating metrics and benchmarks.

## Supplemental Material

sj-docx-1-opp-10.1177_10781552231174858 - Supplemental material for Oncology pharmacy practice in the United States: Results of a comprehensive, nationwide survey Supplemental material, sj-docx-1-opp-10.1177_10781552231174858 for Oncology pharmacy practice in the United States: Results of a comprehensive, nationwide survey by Shawn P Griffin, Jessie R Signorelli, Aubrey Lasko, Benjamin J Andrick, David Doan, Shannon Hough, Grazyna Riebandt and Stephen Harnicar in Journal of Oncology Pharmacy Practice
