# Supplementary material for: Oncology pharmacy practice in the United States: Results of a comprehensive, nationwide survey
Source: J Oncol Pharm Pract. 2023 May 16;30(2):332–41. doi: 10.1177/10781552231174858 (PMC10943602; doi:10.1177/10781552231174858)
Supplement: sj-docx-1-opp-10.1177_10781552231174858 - Supplemental material for Oncology pharmacy practice in the United States: Results of a comprehensive, nationwide survey [file sj-docx-1-opp-10.1177_10781552231174858.docx]

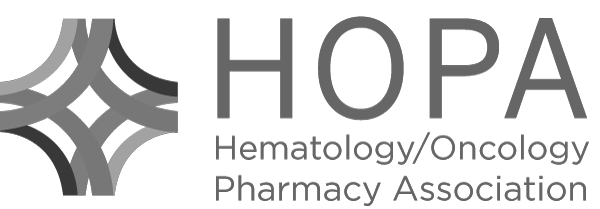


Oncology Practice Landscape Survey

**Welcome to the HOPA Oncology Landscape survey. Your participation will require about 30 minutes and is very much appreciated. You may complete portions of the survey and return to it later, provided you launch from the same device. The survey is divided into pages – if you click “Next” at the bottom of a page, all work completed will be saved.** [**You can download a copy of the survey for reference here.**](https://app.box.com/s/jku3q2zdhdbgmr7iiblfafzgi6yj0b8c)

# 1. Please provide the following information

**Your name**

**Your title**

**The name of your organization**

**The name of your site (if applicable)**

**Email Address**

# * 2. Are you currently a HOPA member?


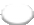

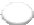
 Yes No


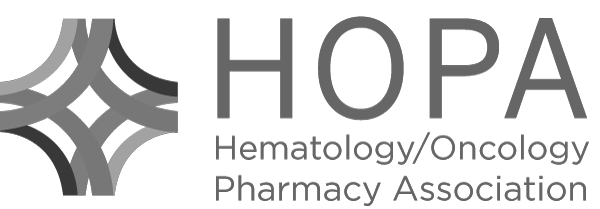


Oncology Practice Landscape Survey

# * 3. Please describe your organization (choose one)


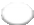
 Academic medical center
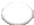
 Community-based center


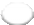
 Accountable care organization
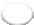
 Other (please specify)

# 4. Of the practice sites offered at your organization, indicate how many of each type provides service to oncology patients.

Main Campus outpatient clinic area

Offsite/satellite clinic(s)

Main Campus Infusion Center

Offsite/satellite Infusion center(s)

Inpatient facility

Retail Pharmacy

Retail Specialty Pharmacy

Home Infusion Services

Other type of practice (please describe and indicate number)

* 5. Who does your highest-ranking pharmacy leader report to? (choose one)
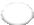
 Chief Executive/Operating Officer (or similar reporting pathway to a business leader)
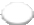
 Chief Medical Officer (or similar reporting pathway to a physician leader)


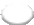
 Chief Nursing Officer (or similar reporting pathway to a nurse leader)


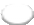
 Board


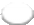
Other (please specify)


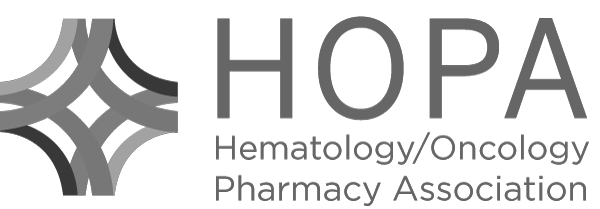


Oncology Practice Landscape Survey

# * 6. Who of the following does your Pharmacy Department train **outside** your Pharmacy Department (choose all that apply)

Medical Students Medical Residents

Oncology or Hematology Fellows Advance Practice Practitioners (APPs) Nursing Students

Nurses

Other (please specify)

# * 7. Who of the following does your Pharmacy Department train **inside** your Pharmacy Department (choose all that apply)

Pharmacy Technicians Pharmacy students and interns PGY1 residents

PGY2 residents Pharmacy fellows

Other (please specify)

# 8. How many positions of all pharmacy residents do you currently have at your organization?

PGY-2 Oncology Residents

PGY-2 Residents (total residents in the second year of their program, including oncology residents)

PGY-1 Residents (total residents in the first year of their training)

# 9. How many ambulatory oncology patient visits does your organization provide each year?

Oncology Providers visits (including medical and surgical oncology, radiation treatment and supportive care visits)

Infusion visits (including non-chemotherapy visits)

# 10. How many total infusion chairs there are in your organization?


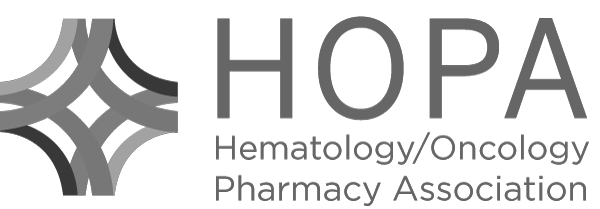


Oncology Practice Landscape Survey

- 11. How many **total** inpatient beds does your organization have?


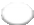
 1-99


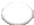
 100-249


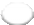
 250-499


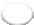
 500-999


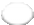
 Over 1000 (please specify)

# 12. How many **dedicated** oncology inpatient beds do you have in your organization?


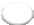
 0-49


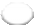
 50-99


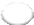
 100-249


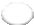
 250-499


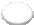
 500-999


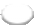
 Over 1000 (please specify)

# * 13. On average, what is the estimated % capacity of your oncology inpatient beds filled?


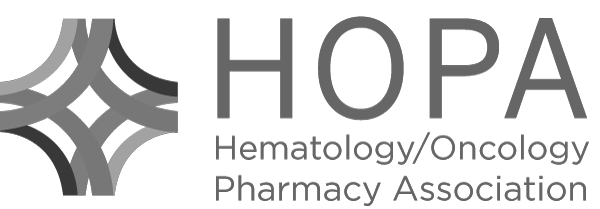


Oncology Practice Landscape Survey

* 14. Please indicate how many **oncology non-pharmacy** practitioners (FTEs) work within your health system for each relevant category (including medical and surgical oncology, radiation treatment and supportive care practitioners)?

Physicians

Advanced Professional Practitioners (APPs)

Nurses

# * 15. Does your organization also employ the following disciplines that care specifically for cancer patients? (choose all that apply)

Medical scribes Patient navigators Case managers Medical assistants

Medication access coordinators Social workers

Dietitians

Research study coordinators Financial advocates

Not applicable

- 16. Please indicate how many **Oncology Pharmacy** (FTEs) work within your organization.
- 17. Of the FTEs in question 16, please answer the following for **inpatient pharmacists.**

**Total** number of pharmacists

% of time / effort / activity to **operations**

% of time / effort / activity to **clinical**

- 18. Of the FTEs in question 16, please answer the following for **ambulatory pharmacists, infusion / clinic.**

**Total** number of pharmacists

% of time / effort / activity to **operations**

% of time / effort / activity to **clinical**

- 19. Of the FTEs in question 16, please answer the following for **pharmacist, retail / specialty.**

**Total** number of pharmacists

% of time / effort / activity to **operations**

% of time / effort / activity to **clinical**

# 20. Of the FTEs in question 16, please answer the following for **IDS.**

**Total** number of pharmacists

% of time / effort / activity to **operations**

% of time / effort / activity to **clinical**

# 21. Of the FTEs in question 16, please answer the following for **technicians.**

**Total** number of technicians

% of time / effort / activity to **operations**

% of time / effort / activity to **clinical**

- 22. Based on your **total oncology pharmacist** FTEs, how many oncology pharmacists in your organization have the following?

BCOP certification

Other BPS certification

PGY1 residency training

PGY2 oncology residency training


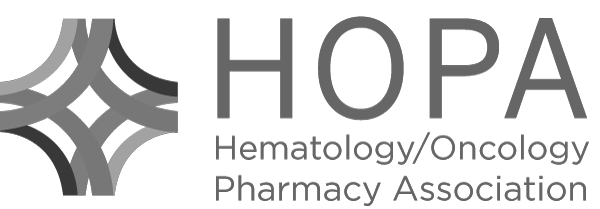


Oncology Practice Landscape Survey

- - 23. Does your **state board of pharmacy** have a credentialing and privileging system for oncology pharmacists?


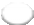
 Yes
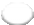
 No

Please describe

# 24. Does your **organization** have a credentialing and privileging system for oncology pharmacists?


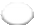
 Yes
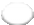
 No

Please describe

# 25. If your organization has a credentialing and privileging system for oncology pharmacists, what **ambulatory setting** privileges are gained through this process? Choose all that apply.

Disease state management Physical exam

Medication safety monitoring Evaluate need and order vaccinations Administer vaccinations

Administer other medications Collaborative Practice Agreements Pharmacy To Dose Protocols Prescriptive Authority

Dose Modifications Ordering labs None

Other (please specify)

# 26. If your organization has a credentialing and privileging system for oncology pharmacists, what **inpatient setting** privileges are gained through this process? Choose all that apply.

Disease state management Physical exam

Medication safety monitoring Evaluate need and order vaccinations Administer vaccinations

Administer other medications Collaborative Practice Agreements Pharmacy To Dose Protocols Prescriptive Authority

Dose Modifications Ordering labs None

Other (please specify)

# 27. Is there a requirement for advanced pharmacy practice certification or other proofs of training or competence at your organization for oncology pharmacist?


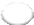
 Certification required
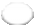
 Certification encouraged


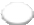
 Not required or encouraged Comment

# 28. If there is a requirement for advanced pharmacy practice certification or other proofs of training or competence at your organization for oncology pharmacists, for which job roles are they required?

PGY2

PGY1 oncology

residency residency

BCOP

Other BPS

Other

Not

training

training

certification certification certification required


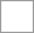

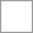

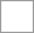

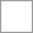

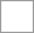

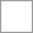


Inpatient pharmacist

Ambulatory pharmacist infusion


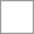

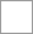

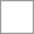

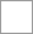

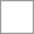

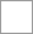


Ambulatory pharmacist retail/specialty

IDS pharmacist


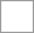

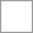

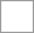

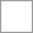

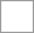

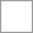


Clinical pharmacist specialist, inpatient

Clinical pharmacist specialist, ambulatory


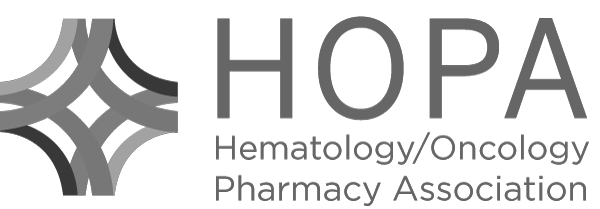


Oncology Practice Landscape Survey

# 29. Are your oncology **pharmacists** involved in any of the following programs?

Drug prior authorizations Patient education

Patient assistance to access oncology drugs Transitional Care

Med Reconciliation

Other (please specify)

# 30. Are your oncology pharmacy **technicians** involved in any of the following programs? Choose all that apply.

Prior drug authorizations

Patient assistance to access oncology drugs Transitional care

Med reconciliation Not applicable

Please describe other and/or provide any comments.


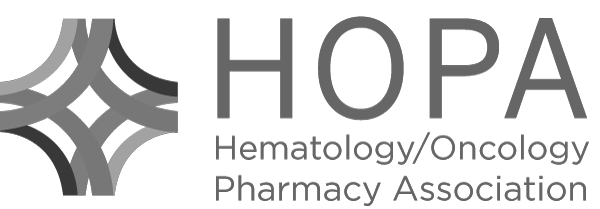


Oncology Practice Landscape Survey

# 31. How would you describe the payment model for your primary practice site at the present time (select all that apply)?

Fee for service

Value-based or outcome-based program Other (please specify)

# 32. Is your organization participating in the oncology care model (OCM) - or other alternative payment model? If yes please indicate which programs.


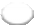
 Yes (please specify below)
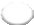
 No

Please specify

# 33. Please indicate the approximate percentage of each type of primary payer for your patients. Your total should equal 100.

Federal: Medicare/Medicaid

Federal: VA/DOD

Commercial

Commercial Medicare/Medicaid

Other
